# Quercitrin from *Toona sinensis* (Juss.) M.Roem. Attenuates Acetaminophen-Induced Acute Liver Toxicity in HepG2 Cells and Mice through Induction of Antioxidant Machinery and Inhibition of Inflammation

**DOI:** 10.3390/nu8070431

**Published:** 2016-07-15

**Authors:** Van-Long Truong, Se-Yeon Ko, Mira Jun, Woo-Sik Jeong

**Affiliations:** 1 Department of Smart Food and Drug, College of Biomedical Science & Engineering, Inje University, Gimhae 50834, Korea; truonglongpro@gmail.com (V.-L.T.); kose91@daum.net (S.-Y.K.); 2 Department of Food and Science & Nutrition, Dong-A University, Busan 49315, Korea; mjun@dau.ac.kr

**Keywords:** quercitrin, acetaminophen, hepatoprotection, Nrf2/ARE, antioxidant

## Abstract

Quercitrin is found in many kinds of vegetables and fruits, and possesses various bioactive properties. The aim of the present study was to elucidate hepatoprotective mechanisms of quercitrin isolated from *Toona sinensis* (Juss.) M.Roem. (syn. *Cedrela sinensis* Juss.), using acetaminophen (APAP)-treated HepG2 cell and animal models. In an in vitro study, quercitrin suppressed the production of reactive oxygen species and enhanced expression of nuclear factor E2-related factor 2 (Nrf2), activity of antioxidant response element (ARE)-reporter gene, and protein levels of NADPH: quinone oxidoreductase 1 (NQO1), catalase (CAT), glutathione peroxidase (GPx), and superoxide dismutase 2 (SOD-2) in APAP-treated HepG2 cells. In an in vivo study, Balb/c mice were orally administered with 10 or 50 mg/kg of quercitrin for 7 days and followed by the injection with single dose of 300 mg/kg APAP. Quercitrin decreased APAP-caused elevation of alanine aminotransferase and aspartate aminotransferase levels, liver necrosis, the expression of pro-inflammatory factors including inducible nitric oxide synthase, cyclooxygenase 2 and inerleukin-1β, and phosphorylation of kinases including c-Jun N-terminal kinase and p38. Quercitrin restored protein levels of Nrf2, NQO1 and activities and expressions of CAT, GPx, SOD-2. The results suggested that quercitrin attenuates APAP-induced liver damage by the activation of defensive genes and the inhibition of pro-inflammatory genes via the suppressions of JNK and p38 signaling.

## 1. Introduction

Acetaminophen (APAP, marketed as Paracetamol^®^ or Tylenol^®^), a widely used over-the-counter analgesic and antipyretic drug, is safe and effective at therapeutic dose. However, it can cause acute liver failures such as serve hepatic necrosis, hepatic lesions and cirrhosis, and even death when taken in high doses. APAP-induced hepatotoxicity is initiated by formation of excess reactive intermediate *N*-acetyl-p-benzoquinone imine (NAPQI), which is produced by metabolism of liver cytochrome P450s (CYPs) including CYP2E1, CYP3A4 and CYP1A2 [1]. NAPQI depletes cellular glutathione (GSH) and adenosine triphosphate (ATP), causes mitochrondrial dysfunction and damage, and finally causes DNA fragmentation and apoptosis. Acetaminophen also contributes to overproduction of reactive oxygen species (ROS) [2]. In addition, lipid peroxidation resulting from oxidative stress has been demonstrated to contribute to the initiation and progression of APAP-induced liver damage [3]. Furthermore, accumulating evidence indicates that acetaminophen overdose triggers the transcriptional activation of pro-inflammatory mediators and cytokines such as inducible nitric oxide (iNOS), cyclooxygenase 2 (COX-2), tumor necrosis factor α (TNF-α), and interleukin 1β (IL-1β) [2,4].

*N*-acetylcysteine (NAC) is used as a clinical antidote for APAP poisoning [5,6]. NAC protects liver from APAP-induced injury by restoring hepatic glutathione and scavenging free radicals [7]. However, NAC therapy has considerable limitations because the therapeutic effect of this drug is quite narrow and it could cause some side-effects such as nausea, anaphylactic reaction, headaches and diarrhea [7,8]. Thus, alternative and effective agents are needed to prevent APAP-induced hepatotoxicity.

**Figure 1:**
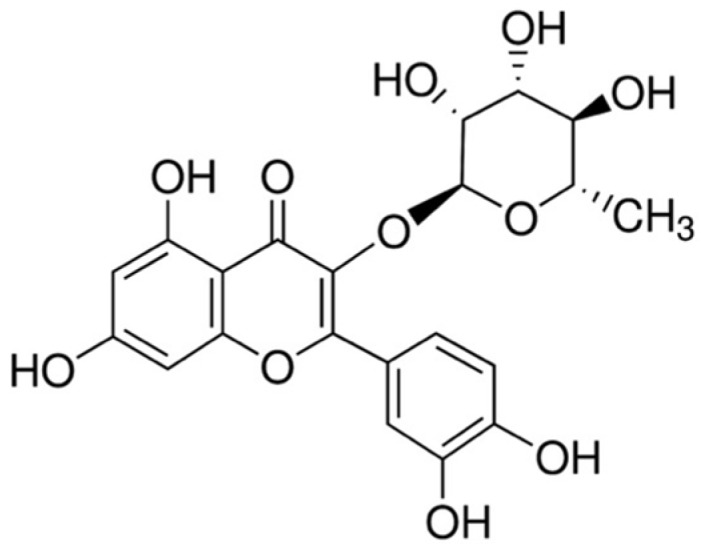
Chemical structure of quercitrin.

*Toona sinensis* (Juss.) M.Roem. (syn. *Cedrela sinensis* Juss.), a member of the Meliaceae family, is an upland tree that has been used as traditional medicine and nutritious food for a long time in China and Korea. *T. sinensis* leaves contain a variety of bioactive compounds such as gallic acid, methyl gallte, kaemferol, quercetin, rutin, quercitrin, palmitic acid and linoleic acid, etc. [9,10,11,12]. Of these compounds, quercitrin (3-rhamnosyl quercetin) (Figure 1), a glycoside of quercetin, has been found as a main bioactive constituent in *T. sinensis* leaves [13,14]. The sugar portion bound to the aglycone in quercitrin has been demonstrated to increase solubility of quercitrin and consequently improve the absorption by interacting with the sodium dependent glucose transport receptor in the small intestine [15,16]. Pharmacological studies have shown that quercitrin has antioxidant [17,18], anti-inflammatory [19,20], and anti-allergic [21] activities. Quercitrin has been demonstrated to exert a protective effect against UV-induced cell death and apoptosis [22,23]. Furthermore, quercitrin has been studied as an anti-cancer agent against non-small cell lung cancer through the modulation of immune response [24]. Our group previously reported that quercitrin from *T. sinensis* leaves potently scavenged free radicals and induced antioxidant enzymes in H_2_O_2_-treated HepG2 cells [9]. However, the protective mechanisms of quercitrin against APAP-induced hepatotoxicity have not been investigated.

In the present study, we examined the hepatoprotective effects of quercitrin in both cell line and animal models of hepatotoxicity challenged by APAP. The stimulating activity of quercitrin on cellular defensive genes including transcription factor nuclear factor E2-related factor 2 (Nrf2), antioxidant response element (ARE)-reporter gene and antioxidant enzymes was determined. The inhibitory effects of quercitrin on liver damage markers of alanine aminotransferase (ALT) and aspartate aminotransferase (AST) as well as inflammatory targets of iNOS, COX-2 and IL-1β were evaluated in APAP-induced hepatotoxic mice. In addition, the modulation of mitogen activated protein kinases (MAPKs) by quercitrin was examined.

## 2. Materials and Methods

### 2.1. Chemicals

Acetaminophen, silymarin, MTT [3-(4,5-dimethylthiazol-2-yl)-2,5-diphenyltetrazolium bromide], dimethyl sulphoxide (DMSO), polyethylene glycol (PEG), and triton X-100 were purchased from Sigma-Aldrich (St. Louis, MO, USA). 2′,7′-dichlorodihydrofluorescein diacetate (DCFH-DA) was purchased from Molecular Probe Inc. (Eugene, OR, USA). The colorimetric ALT and AST assay kits were supplied by Young-Dong Co. (Seoul, Korea). Anti-Nrf2 (sc-13032), anti-NQO1 (sc-25591), anti-SOD-2 (sc-18504), anti-GPx (sc-30147), anti-COX-2 (sc-1745), anti-iNOS (sc-651), anti-IL-1β (sc-7884), anti-β-actin (sc-1616), and horseradish peroxidase-conjugated anti-goat immunoglobulin IgG (sc-2350) antibodies were purchased from Santa Cruz Biotechnology Inc. (Santa Cruz, CA, USA). Anti-p-ERK (9101), anti-p-JNK (9251), anti-p-p38 MAPK (9211), anti-CAT (14097), and horseradish peroxidase-conjugated anti-rabbit immunoglobulin IgG (7074) were purchased from Cell Signaling Technology (Beverly, MA, USA). All other reagents used in this study were of the highest grade commercially available.

### 2.2. Preparation of Quercitrin

Quercitrin from powder of *T. sinensis* (Juss.) M.Roem. leaves, kindly provided by Slow-life Chamjook Farm Corporation (Hamyang, Korea), was isolated according to previous literature [9]. A voucher specimen (Jeong, W.S. 001) of the raw material was deposited at the Herbarium of Kyungpook National University (KNU), Daegu, Korea. Briefly, the powder of *T. sinensis* was extracted three times with 95% ethanol (EtOH) for 24 h in room temperature and concentrated by rotary evaporation at 40 °C. After concentration, the concentrate was suspended in 10% EtOH and then fractionated with a series of organic solvents, including h-hexane, DCM, ethylacetate (EtOAc), n-butanol, and water in sequence. The EtOAc-solution fraction, the most potent antioxidant fraction to both scavenging radicals and antioxidant enzyme activity, was applied onto a silica gel open column. The column was eluted with solvent mixtures of DCM/methanol under gradient conditions to yield 17 fractions. The most potent subfraction (Fr. 10) was purified by recycling preparatory high-performance liquid chromatography (HPLC) and structural analyses were performed using 1H- and 13C-nuclear magnetic resonance (NMR) analysis.

### 2.3. Cell Culture

Human hepatoma cell line HepG2, obtained from American Type Culture Collection (ATCC, Rockville, MD, USA), was maintained DMEM containing 10% fetal bovine serum (FBS), 100 units/mL penicillin, 100 µg/mL streptomycin, 1% essential amino acids, 1% glutamax in humidified atmosphere of 5% CO_2_ at 37 °C. The medium was renewed once every two days.

### 2.4. Cell Viability Assay

Cell viability was examined using MTT assay. Briefly, HepG2 cells were cultured in a 24-well plate at density of 1 × 10^5^ cells/well. After starvation, cells were treated with a series of various concentrations of quercitrin in the presence of 10 mM APAP for 24 h and then incubated with 5 mg/mL MTT for additional 4 h. The dark blue formazan crystal was solubilized in DMSO and followed by measurement of absorbance at 570 nm with a microplate reader (BioTek Instruments, Winooski, VT, USA).

### 2.5. ROS Formation Assay

Level of intracellular ROS was measured using DCFH-DA. In viable cells, the DCFH-DA is cleaved by esterase to DCFH and followed oxidizing by ROS to form a highly florescent molecule, DCF. Briefly, HepG2 cells were seeded onto 96-well plates at density of 2 × 10^4^ cells/well and then treated either vehicle (DMSO, 0.1%) or various concentrations of quercitrin in the presence of 10 mM APAP for 24 h. After washing twice with phosphate-buffered saline (PBS), the cells were incubated with 20 µM DCF-DA for 1 h at 37 °C in darkness. The fluorescence that is representative of intracellular reactive oxygen species level was measured at 485/20 nm excitation and 528/20 nm emission using a fluorescence multidetection reader (Synergy HT, BioTek, VT, USA).

### 2.6. Assay of Reporter Gene Activity

HepG2-C8 cell line, kindly donated by Ah-Ng Tony Kong (Rutgers University, New Brunswick, NJ, USA), was established by the table transfection of HepG2 cells with p-ARE-T1-luciferase reporter gene as previously described [25]. After starvation, the HepG2-C8 cells were treated with various concentrations of quercitrin for 24 h and then ARE-luciferase activity was measured using a luciferase kit from Promega (Promega Corp., Madison, WI, USA) according to the manufacturer’s instructions. Luciferase activity was normalized against total protein concentration, determined by a BCA protein assay kit (Pierce, Rockford, IL, USA), and expressed as a fold induction over that in vehicle-treated cells.

### 2.7. Animals and Experimental Design

Thirty male Balb/c mice (6 weeks old, 20–25 g) were obtained from Hyochang Science (Daegu, Korea) and allowed free access to standard laboratory diet and distilled water *ad libitum* for at least one week prior to the experiment. The animal room was environmentally controlled at 25 ± 2 °C, 55%–60% humidity, 12 h light/dark cycle. This animal experiment was approved by the Animal Care and Used Committee of Inje University with protocol number 2013-50 (Gimhae, Korea). The mice were randomly divided into six groups with five mice each. Group I (control group) received 50% PEG as a vehicle for 7 days. Group II (quercitrin group) was only administered 50 mg/kg quercitrin for 7 days. Group III (APAP group) received 50% PEG for 7 days. Group IV and V (quercitrin + APAP group) were orally gavaged with 10 or 50 mg/kg of quercitrin, respectively, for 7 consecutive days. Group VI (APAP + silymarin group) was orally administered 50 mg/kg of silymarin (SIL), which is frequently used as a natural hepatoprotectant by its antioxidant and anti-inflammatory properties [26] for 7 days. After 1 h last administration, a single dose of APAP (300 mg/kg) was injected to mice in groups III-VI. After 24 h APAP challenge, all mice were sacrificed to obtain blood and livers for further biochemical analysis.

### 2.8. Measurement of Plasma ALT and AST Levels

Blood samples from mice were centrifuged at 3000× *g*, 4 °C for 10 min to obtain serum supernatant. The ALT and AST activities in serum were measured using commercially available kits (Young-dong Pharm, Seoul, Korea).

### 2.9. Assay for Antioxidant Enzymes Activity

Liver tissues were collected and homogenized in phosphate buffer (pH 7.0) and then tissue homogenates were centrifuged at 10,000× *g*, 4 °C for 10 min to obtain separately supernatant and pellet for further experiments. The protein concentration was determined using a BCA protein assay kit (Pierce, Rockford, IL, USA). CAT activity was determined by the method of Aebi [27]. GPx activity was performed according to the method of Bongdanska et al. [28]. SOD activity was determined by the method of Oyanagui [29]. Enzyme activities were expressed in relation to the vehicle-treated control.

### 2.10. Western Blot Analysis

Cells and liver tissues were homogenized in RIPA buffer and then centrifuged at 10,000× *g*, 4 °C for 10 min to obtain supernatant. Total protein concentration of each sample was determined using a BCA protein assay kit (Pierce, Rockford, IL, USA) according to the manufacturer’s instructions. Equal amounts of protein were resolved on SDS-PAGE gels and then transferred onto PVDF membranes (Millipore, Bedford, MA, USA) using a semidry transfer system (Bio-rad, Hercules, CA, USA). After blocking with 5% non-fat milk in PBST (0.1% Tween 20 in PBS) for 2 h at 4 °C, the membranes were incubated overnight with appropriate primary antibodies at 4 °C. Then, the membranes were washed five times with PBST and hybridized with horseradish peroxidase-conjugated anti-rabbit or anti-goat immunoglobulin IgG secondary antibodies for 3 h at 4 °C. After washing five times with PBST, blots were visualized using enhanced chemiluminescence western blotting reagent (Santa Cruz Biotechnology, Santa Cruz, CA, USA).

### 2.11. Statistical Analysis

Data were expressed as means ± SD. Statistical comparisons were evaluated using analysis of variance (ANOVA) followed by Tukey’s test. *p* < 0.05 was considered as significant.

## 3. Results

### 3.1. Protective Effect of Quercitrin against Oxidative Stress in APAP-Induced HepG2 Cells

**Figure 2:**
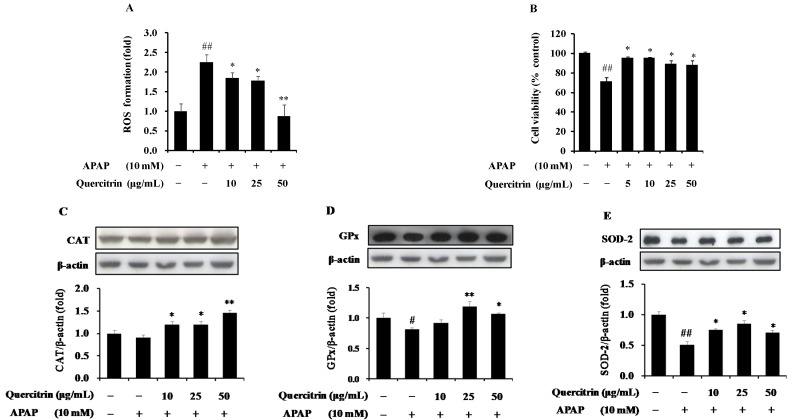
Protective effects of quercitrin on acetaminophen (APAP)-treated HepG2 cells. (**A**) Effect of quercitrin on APAP-mediated reactive oxygen species (ROS) formation; (**B**) Effect of quercitrin on cell viability using MTT assay at 24 h. Effect of quercitrin on protein expressions of (**C**) catalase (CAT); (**D**) glutathione peroxidase (GPx); and (**E**) superoxide dismutase 2 (SOD-2) in APAP-treated HepG2 cells. ROS was presented as the fold induction of vehicle-treated control. Cell viability was normalized as 100% for control (without any treatments). Protein expression levels were normalized with β-actin. All data represent means ± SD of at least three independent experiments. Significant differences were (^##^) *p* < 0.01 or (^#^) *p* < 0.05 as compared with the control; (**) *p* < 0.01 or (*) *p* < 0.05 as compared with the APAP group.

To investigate the effect of quercitrin on the APAP-induced ROS formation, we measured the intracellular level of ROS using a redox-sensitive fluorescence reagent, DCF-DA. As shown in Figure 2A, exposure of HepG2 cells to APAP remarkably elevated the ROS production by over 2-fold at 24 h when compared to the control. Pre-treatment of quercitrin, however, inhibited the increase of APAP-induced ROS level. The presence of 10, 25, 50 µg/mL quercitrin significantly decreased the ROS formation in HepG2 cells by 0.5-, 0.5-, and 1.3-fold, respectively, compared to APAP-treated cells alone. Enhanced oxidative stress can result in apoptosis and cell death; thus, we tested whether quercitrin rescue APAP-induced cell death using MTT assay. Consequently, 10 mM APAP reduced 30% cell viability, which was restored by quercitrin (Figure 2B).

Antioxidant enzymes including CAT, GPx, and SOD constitute the primary part of enzymatic antioxidant defense system against oxidative stress via directly eliminating ROS. Thus, the effects of quercitrin on expression levels of antioxidant enzymes were examined. The results showed that APAP significantly reduced protein expressions of GPx and SOD but not CAT compared with control (Figure 2C–E). However, pretreatment with quercitrin for 1 h blocked the effect of APAP on the decreased expression of antioxidant enzymes. Overall, these results obviously suggested that quercitrin prevents APAP-induced ROS formation and cell death through the induction of antioxidant enzymes in HepG2 cells.

### 3.2. Upregulation of Nrf2/ARE-Mediated Phase II Detoxifying Enzymes by Quercitrin in APAP-Treated HepG2 Cells

**Figure 3:**
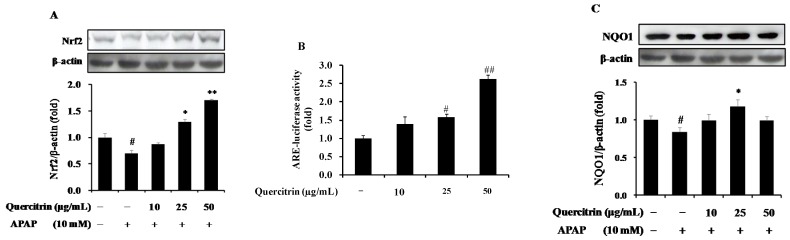
Up-regulation of nuclear factor E2-related factor 2 (Nrf2)/antioxidant response element (ARE)-mediated phase II detoxifying enzyme in APAP-treated HepG2 cells. (**A**) Effect of quercitrin on protein expression of Nrf2; (**B**) Effect of quercitrin on ARE-luciferase activity; (**C**) Effect of quercitrin on protein level of quinone oxidoreductase 1 (NQO1). Luciferase activity was normalized with total protein content and expressed as fold induction of normal control. Protein expression levels were normalized with β-actin. All data represent means ± SD of at least three independent experiments. Significant differences were (^##^) *p* < 0.01 or (^#^) *p* < 0.05 as compared with the control; (**) *p* < 0.01 or (*) *p* < 0.05 as compared with APAP group.

To further confirm the role of quercitrin in protection of cells from APAP toxicity, the activation of Nrf2/ARE pathway was evaluated. Nrf2 is considered as a key transcriptional factor responsible for the regulation of phase II detoxifying and/or antioxidant enzymes, which protect cells from cell death-induced APAP toxicity. The basal levels of protein expression of Nrf2 were detected in HepG2 cells. Compared with untreated control, APAP was able to significantly decrease the Nrf2 protein level for up to 24 h (Figure 3A). Further, we investigated the inhibitory effect of quercitrin on APAP-induced the reduction of Nrf2 expression. Results showed that quercitrin has the potential to induce Nrf2 for 24 h. The quercitrin concentration at ≥25 µg/mL caused a significant increase in protein level of Nrf2. In particular, quercitrin concentration at 50 µg/mL resulted in a strong up-regulation of Nrf2 at 24 h by 1.6-fold compared to control and 2.0-fold compared with APAP alone. In addition, in order to elucidate whether the activation of Nrf2 induces ARE-mediated phase II detoxifying and/or antioxidant enzymes using ARE-reporter gene activity assay, HepG2-C8 cells, stably transfected with a pARE-TI-luciferase reporter gene were exposed to quercitrin for 24 h. As illustrated in Figure 3B, quercitrin enhanced the ARE-luciferase activity. The luciferase activity by quercitrin at the highest concentration of 50 µg/mL reached 2.6-fold compared with that by vehicle-treated control. This result demonstrated the role of Nrf2 in up-regulation of ARE-mediated phase II enzymes. Furthermore, the effect of quercitrin on protein level of a phase II detoxifying enzyme, NQO1, was examined in APAP-treated HepG2 cells. The result showed that treatment with APAP resulted in the decrease of NQO1 protein level; however, pre-treatment of quercitrin restored the APAP-reduced expression level of NQO1, even more than in the control at quercitrin concentration of 25 µg/mL (Figure 3C). These data suggest that quercitrin promotes hepatoprotective system through the activation of Nrf2 and Nrf2/ARE-mediated antioxidant defense mechanism.

### 3.3. Protective Effect of Quercitrin against APAP-Induced Hepatotoxicity in Vivo

**Figure 4:**
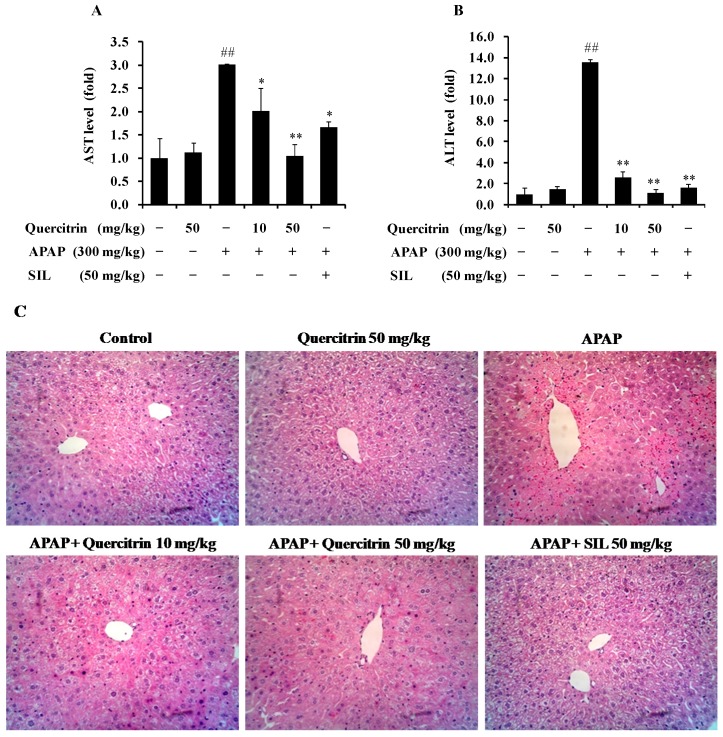
Protective effects of quercitrin on APAP intoxicated mice. Mice were orally administered quercitrin of 10 or 50 mg/kg once a day for seven days. The control group received the same volume of saline. After the seventh gavage of quercitrin or saline, animals received a single injection of APAP (300 mg/kg). All mice were sacrificed at 24 h after APAP injection to obtain blood and liver. Serum ALT (**A**) and AST (**B**) levels; and H&E staining (**C**). All data represent means ± SD of five mice in each group. Significant differences were (^##^) *p* < 0.01 as compared with the control; (**) *p* < 0.01 or (*) *p* < 0.05 as compared with APAP group.

To further investigate the role of quercitrin in protecting liver from APAP-induced toxicity, Balb/c mice were administered quercitrin at a dose of 10 and 50 mg/kg before injection of 300 mg/kg APAP. As shown in Table 1, APAP significantly increased liver weight within 24 h compared with the control and quercitrin alone groups, whereas body weights were comparable among all experimental groups. Oral pre-administration of quercitrin (10 and 50 mg/kg) or SIL (50 mg/kg) reduced the APAP-induced increase of liver weight. In addition, injection of 300 mg/kg APAP highly elevated 14- and 3-fold of serum ALT and AST activities, respectively, compared to the vehicle-treated group, implying that a high dose of APAP caused liver toxicity (Figure 4A,B). Quercitrin treatment significantly prevented the APAP-induced elevation of ALT and AST levels. Similarly, 50 mg/kg of SIL, considered as a positive control in this study, was found to attenuate the ALT and AST activities. Further, the administration of 50 mg/kg quercitrin alone was unlikely to significantly alter the basal activity of these transaminases, suggesting no liver damage caused by quercitrin. Histological (H&E) analysis further revealed that APAP-induced severe hepatic lesions such as hydropic degeneration, inflammation, and hemorrhage were improved by quercitrin pretreatment. Also, a similar result was observed in the positive control group, SIL (Figure 4C). These results prove that quercitrin may be a potential agent in the prevention of APAP-induced liver injury in a mouse model.

### 3.4. Protective Effect of Quercitrin against APAP-Induced Hepatotoxicity through Enhancement of Activity and Expression of Antioxidant Enzymes

**Figure 5:**
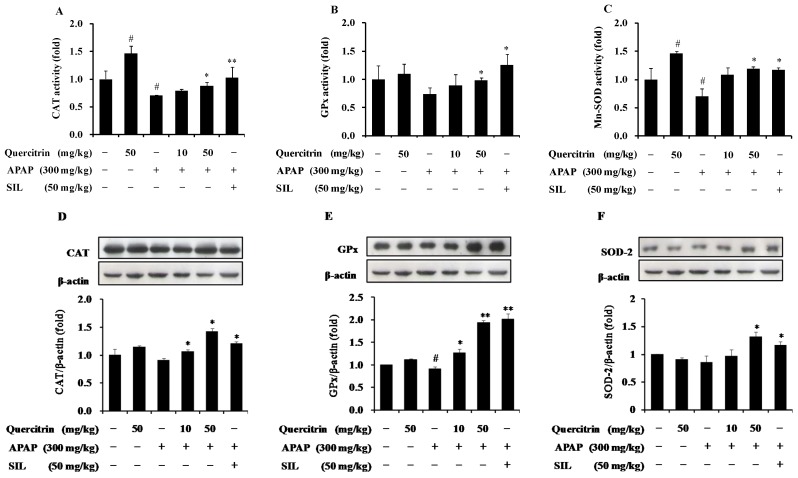
Effects of quercitrin on activities and protein expressions of hepatic antioxidant enzymes in APAP intoxicated mice. The hepatic activities of CAT (**A**); GPx (**B**) and SOD-2 (**C**) were measured as described in Materials and Methods section. The protein levels of CAT (**D**); GPx (**E**) and SOD-2 (**F**) were determined using Western blot analysis. Protein expression levels were normalized with β-actin. All data represent means ± SD of five mice in each group. Significant differences were (^#^) *p* < 0.05 as compared with the control; (**) *p* < 0.01 or (*) *p* < 0.05 as compared with APAP group.

To examine whether quercitrin activates antioxidant defense system in APAP-treated mouse models, we measured activity and protein level of antioxidant enzymes including CAT, GPx, and SOD-2, which importantly contribute to the protection of cells/tissues from oxidative stress through directly neutralizing and eliminating ROS. The results indicated that injection of mice to 300 mg/kg APAP resulted in the reduction in activities of three antioxidant enzymes. Quercitrin pre-administration, however, prevented the APAP-induced decrease of CAT, GPx, and SOD-2 enzyme activities (Figure 5A–C). It is likely that quercitrin, SIL (50 mg/kg), also significantly increased the activities of these enzymes when compared to APAP-treated mice. Interestingly, oral administration of 50 mg/kg quercitrin alone was shown to enhance the CAT and SOD-2 activities but not GPx activity compared to the vehicle-treated control. In order to further confirm the inductive effect as well as the role of quercitrin on liver protection against APAP-induced oxidative stress, protein expression levels of antioxidant enzymes including CAT, GPx, and SOD-2 were determined. The results showed that treatment of 300 mg/kg APAP caused slightly reduced protein expressions of these antioxidant enzymes. However, as expected, quercitrin significantly induced the APAP-abolished CAT, GPx, and SOD-2 proteins, even more than those in the control group (Figure 5D–F). This result is consistent with SIL, which also enhanced the expressions of antioxidant enzymes in APAP-treated mice. Overall, our study supports the use of quercitrin in preventing APAP-mediated liver toxicity.

### 3.5. Upregulation of NQO1 Expression by Nrf2-Mediated Quercitrin in APAP-Treated Mice

**Figure 6:**
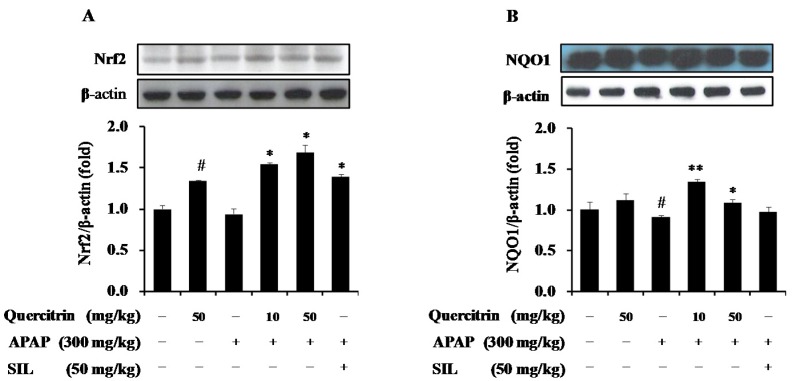
Effects of quercitrin on expressions of Nrf2 and NQO1 in APAP intoxicated mice. The protein levels of Nrf2 (**A**) and NQO1 (**B**) were measured by Western blot analysis as described in Materials and Methods section. Protein expression levels were normalized with β-actin. All data represent means ± SD of five mice in each group. Significant differences were (^#^) *p* < 0.05 as compared with the control; (**) *p* < 0.01 or (*) *p* < 0.05 as compared with APAP group.

As demonstrated, quercitrin up-regulated Nrf2-mediated phase II detoxifying enzyme in HepG2 cells, thus we confirmed the inductive effect of quercitrin on protein levels of NQO1 in an APAP-treated mouse model. Similar to the in vitro result, exposure of mice to APAP caused a significant attenuate of NQO1 protein level (Figure 6B) but was unlikely to suppress protein expression of Nrf2 after 24 h challenge (Figure 6A) when compared to the untreated control. The presence of quercitrin restored the APAP-reduced protein expression of both Nrf2 and NQO1. Further, the levels of Nrf2 and NQO1 expression induced by quercitrin were even higher than those induced by a positive control, SIL (50 mg/kg) (Figure 6A,B). These results demonstrate the role of quercitrin in the activation of Nrf2/ARE-mediated phase II detoxifying enzyme, implying protection of cells/tissues against APAP-induced toxicity.

### 3.6. Quercitrin Attenuates Inflammatory Response in APAP-Treated Mice

**Figure 7:**
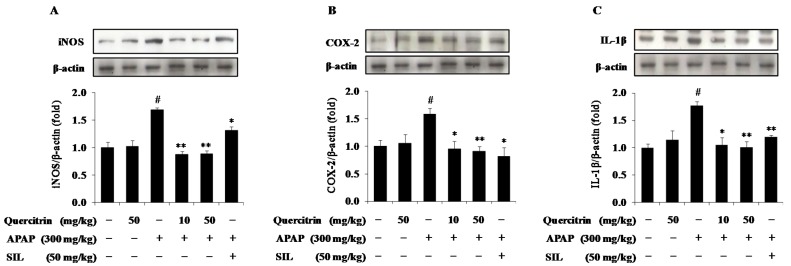
Effects of quercitrin on inflammatory response in APAP intoxicated mice. The hepatic inducible nitric oxide (iNOS) (**A**); cyclooxygenase 2 (COX-2) (**B**) and interleukin 1β (IL-1β) (**C**) levels were evaluated by Western blot analysis. Protein expression levels were normalized with β-actin. All data represent means ± SD of five mice in each group. Significant differences were (^#^) *p* < 0.05 as compared with the control; (**) *p* < 0.01 or (*) *p* < 0.05 as compared with APAP group.

In further studies, we evaluated the effects of quercitrin on APAP-induced expression of pro-inflammatory mediators and cytokines implicated in hepatotoxicity. APAP treatment significantly increased protein expression for the pro-inflammatory mediators and cytokine such as iNOS, COX-2 and IL-1β compared to vehicle-treated control, whereas animals receiving quercitrin alone did not show elevated levels of these hepatic inflammatory factors (Figure 7A–C). APAP-enhanced protein expression of iNOS, the enzyme mediating production of nitric oxide, was also inhibited by the presence of quercitrin (Figure 7A). Expression of pro-inflammatory mediator COX-2 that was up-regulated in mice exposing APAP intoxication was reduced in mice pre-treated with quercitrin (Figure 7B). Additionally, quercitrin administration resulted in a decrease in the APAP-induced IL-1β protein level (Figure 7C).

### 3.7. Activation of Cytoprotective Mechanisms by Quercitrin through Regulation of MAPK Pathways in APAP-Treated Mice

**Figure 8:**
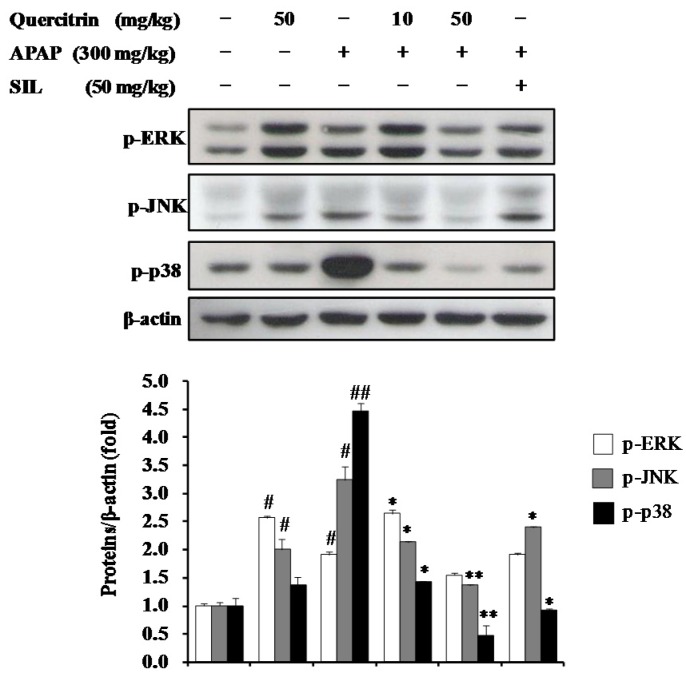
Effects of quercitrin on MAP kinases in APAP intoxicated mice. The phosphorylations of ERK, JNK and p38 MAPK were analyzed by Western blotting. Protein expression levels were normalized with β-actin. All data represent means ± SD of five mice in each group. Significant differences were (^##^) *p* < 0.01 or (^#^) *p* < 0.05 as compared with the control; (**) *p* < 0.01 or (*) *p* < 0.05 as compared with APAP group.

To investigate role of quercitrin on the modulation of MAPKs in APAP-treated mice, phosphorylation levels of MAPKs including ERK, JNK, and p38 MAPK were determined using western blotting. As shown in Figure 8, exposure of APAP (300 mg/kg) increased the phosphorylation levels of ERK, JNK, and p38 MAPK in the liver by 2-, 3-, and 4-fold, respectively, when compared to the control group. This suggested that MAPKs involved in APAP-caused hepatotoxicity. On other hand, treatment of quercitrin (50 mg/kg) alone resulted in a significant elevation of phosphorylated ERK and JNK but not phosphorylated p38. Oral administration of quercitrin or SIL, however, strongly inhibited the JNK and p38 MAPK phosphorylations, whereas ERK phosphorylation was slightly decreased at 50 mg/kg quercitrin and there was no change at 50 mg/kg SIL. Overall, our results indicate that the inhibition of APAP-induced phosphorylation levels of JNK and p38 MAPK by quercitrin contributes to protection of liver from toxicity.

## 4. Discussion

The present study demonstrated the role of quercitrin from *T. sinensis* in the protection of cells/tissues from APAP-induced toxicity. Quercitrin attenuated the ROS formation and cell death induced by APAP overdose through the restoration of antioxidant enzymes as well as enhancement of Nrf2/ARE pathway-mediated cytoprotecive enzymes in HepG2 cells. Moreover, oral administration of quercitrin exhibited protective effects against APAP-caused liver injury in mice, which is consistent with an in vitro test. Indeed, quercitrin pretreatment prevented APAP toxicity, as evidenced by much less liver injury and decreased serum ALT and AST levels. Quercitrin restored the APAP-induced reduction of activities and protein expressions of antioxidant enzymes, including CAT, GPx, and SOD-2 as well as protein level of phase II enzyme NQO1 through the activation of transcription factor Nrf2. In addition, quercitrin exerted the anti-inflammatory property through inhibiting the expression of pro-inflammatory mediators and cytokine such as iNOS, COX-2, IL-1β in APAP-treated liver. Furthermore, inhibition of APAP-mediated phosphorylation levels of JNK and p38 MAPK were considered as a protective mechanism of quercitrin from APAP toxicity in liver. These results, for the first time, demonstrate that antioxidant and anti-inflammatory properties of quercitrin potently contribute to the prevention of APAP-induced liver injury.

Reactive oxygen species (ROS) including free radicals such as superoxide (O_2_·^−^), hydroxyl radical (·OH), peroxyl radical (RO_2_·) and non-radical species such as hydrogen peroxide (H_2_O_2_) are implicated in the development of various diseases such as aging, cancer, atherosclerosis, fibrosis, and inflammation [30,31,32]. The accumulation of ROS results in oxidative stress, lipid peroxidation, protein inactivation, DNA damage, loss of cell function, and finally apoptosis and necrosis [33]. Increased oxidative stress and massive impairment of antioxidant defense systems are well-known as the main mechanisms of APAP-caused hepatotoxicity [34]. The major reactive oxygen species generated by APAP toxicity is superoxide (O_2_·^−^), which dismutates to molecular oxygen and hydrogen peroxide or reacts with nitric oxide (NO) to produce peroxynitrite (ONOO^−^), a potent oxidant and nitrating species [35]. A variety of phenomena such as lipid peroxidation, increased ALT and AST, reduced levels of cellular antioxidant enzymes, and DNA damages was observed in APAP toxicity. In this study, we confirmed that ROS levels were dramatically increased, whereas protein expressions and activities of CAT, GPx, and SOD-2 in HepG2 cells and mouse livers significantly decreased after APAP treatment. In addition, HepG2 cell death and liver necrosis were elevated, suggesting that APAP-induced ROS formation results in loss of cell function and ultimately apoptosis. However, quercitrin could inhibit ROS formation and consequently reduce hepatic cell death as well as liver necrosis caused by APAP toxicity. Quercitrin also remarkably abolished plasma ALT and AST levels, which coincided with the decrease of APAP-mediated oxidative damage. Furthermore, the levels of antioxidant enzymes including CAT, GPx, and SOD-2 were restored by pretreatment of quercitrin in APAP-treated HepG2 cells and livers of mice. These results indicate that quercitrin protects cells/livers from APAP-induced oxidative stress through the suppression of ROS production and enhancement of an antioxidant defense system.

Recent studies have demonstrated that transcription factor Nrf2 importantly contributes to cellular defense mechanisms from APAP toxicity [36,37]. Treatment of Nrf2 knockout mice (Nrf2-/-) with APAP results in enhanced hepatotoxicity and mortality compared to wild-type [36]. NAC treatment produced a low efficacy in Nrf2-/- mice, although NAC still exhibited the effective protection against APAP toxicity in wild-type mice [37,38]. Additionally, hepatocyte-specific Kelch-like ECH-associated protein-1 (*keap1*) gene knockout mice that highly express detoxifying enzymes are significantly more resistant to APAP toxicity than wild-type animals [39]. Consequently, up-regulation of phase II antioxidant/detoxifying enzymes by Nrf2/ARE pathway plays a critical role in preventing hepatic damage caused by APAP. To clarify the underlying hepatoprotective mechanism of quercitrin, we investigated the inductive effect of quercitrin on the Nrf2/ARE pathway. The results suggested that protein level of Nrf2 was enhanced by the pre-treatment of quercitrin in both in vitro and in vivo models. Quercitrin also significantly increased the ARE-reporter gene activity in HepG2-C8 cells. Furthermore, quercitrin restored the APAP-induced reduction of phase II enzyme NQO1, which are capable of scavenging oxidative stress and detoxifying/eliminating toxic chemicals. NQO1 has been demonstrated to be capable of lowering NAPQI availability by the conversion of NAPQI back to the parent APAP, prevent adenosine triphosphate (ATP) depletion as well as mitochondrial dysfunction caused by APAP [40,41]. Our findings strongly indicate that quercitrin enhances the hepatic defense system through the activation of Nrf2/ARE-mediated phase II detoxifying/ antioxidant enzymes.

Uncontrolled inflammation initiates the progression of tissue damage and results in the development of many diseases such as Alzheimer’s disease, cardiovascular disease, atherosclerosis and cancer [42,43]. During the inflammatory response, pro-inflammatory mediators and cytokines such as iNOS, COX-2, IL-1β, and TNF-α were up-regulated and accumulated in the liver [44]. Numerous evidence has showed that APAP causes early damage, which triggers an inflammatory response with the production of pro-inflammatory factors and innate immune cell infiltration in the liver [45,46,47,48]. Thus, inhibition of these mediators and cytokines production is an important mechanism for treatment of inflammation, contributing to preventing APAP-induced hepatotoxicity. In the current study, we confirmed that APAP intoxication resulted in increased expression levels of iNOS, COX-2 as well as IL-1β in the liver. However, mice pre-administered quercitrin exhibited the inhibitory effect on protein expressions of iNOS, COX-2, and IL-1β. These results, for the first time, imply that the hepatoprotective effect of quercitrin may be due to its anti-inflammatory property.

MAP kinases including ERK, JNK, and p38 MAPK play a crucial role in transduction extracellular signals to cellular responses including cellular proliferation, differentiation, development, inflammation and apoptosis in response to various signals produced by growth factors, hormones, cytokines, and xenobiotics as well as oxidative stress [49,50]. A massive study demonstrated that MAP kinases directly implicate in the mechanism of APAP-caused hepatocellular injury. During APAP overdose, JNK is phosphorylated and translocates into mitochondria early in APAP hepatotoxicity in mice, resulting in an initial oxidative stress, induction of mitochorial permeability transition, and inhibition of bioenergetics [35,51]. Additionally, activated JNK can trigger Bax activation, which is responsible for the initial apoptosis-inducing factor (AIF) and endonuclease G release from the mitochondria and early DNA damage, as well as induce iNOS expression, which promotes peroxynitrite formation [52,53]. Furthermore, inhibition of JNK activation or *jnk* gene knockout in mice decreased the liver injury, nuclear DNA fragmentation, and prevented the development of mitochondrial oxidative stress after APAP overdose [51,52,54]. Different from JNK inhibitor, the inhibitors of ERK and p38 MAPK exhibited no protection against APAP toxicity either in vitro or in vivo [52]. Although activations of ERK and p38 MAPK were observed, the roles of ERK and p38 MAPK in APAP-caused heptotoxic mechanism have not been clearly investigated. Overall, the inhibition of JNK phosphorylation contributes to reduction of liver injury induced by APAP challenge. In the present study, we also investigated the inhibition of JNK phosphorylation and concluded it could play a part in the hepatoprotective mechanism of quercitrin against APAP-mediated damage.

## 5. Conclusions

In conclusion, quercitrin protects against APAP-induced liver injury by enhancing the cellular defense system and lowering inflammatory response. Activation of Nrf2/ARE pathway-mediated cytoprotective proteins by quercitrin may contribute to neutralizing ROS, eliminating toxicants and finally preventing cell death and liver necrosis. Quercitrin also exhibits anti-inflammatory properties through the inhibition of pro-inflammatory mediators including iNOS and COX-2 as well as a cytokine IL-1β in APAP-caused inflammation. Furthermore, the suppression of JNK phosphorylation by quercitrin was identified as a protective mechanism against apoptosis. Our study revealed that quercitrin may have safe therapeutic potential in protecting cell/liver from APAP toxicity.

## Figures and Tables

**Table 1:** Effect of quercitrin on body weight and liver weight/body weight (%) in APAP intoxicated mice.

Groups	Treatment	Initial Body Weight (g)	Final Body Weight (g)	Liver Weight (g)	Relative Liver Weight (Percentage Ratio)
I	Control	20.12 ± 0.49	23.02 ± 1.36	1.42 ± 0.09	6.19 ± 0.30
II	Quercitrin (50 mg/kg)	19.62 ± 0.59	23.40 ± 0.45	1.53 ± 0.11	6.55 ± 0.53
III	APAP (300 mg/kg)	19.68 ± 0.40	23.37 ± 0.90	1.65 ± 0.11	7.21 ± 0.36 ^#^
IV	Quercitrin (10 mg/kg) + APAP	20.20 ± 0.34	24.1 ± 0.41	1.50 ± 0.10	6.20 ± 0.31 *
V	Quercitrin (50 mg/kg) + APAP	19.92 ± 0.38	23.02 ± 0.97	1.47 ± 0.09	6.40 ± 0.28 *
VI	Silymarin (50 mg/kg) + APAP	19.68 ± 0.85	23.42 ± 0.92	1.50 ± 0.10	6.38 ± 0.19 *

(^#^) *p* < 0.05 as compared with the control, (*) *p* < 0.05 as compared with APAP group.

## Abbreviations

The following abbreviations are used in this manuscript:

APAP	Acetaminophen
ALT	Alanine transaminase
ARE	Antioxidant response element
AST	Aspartate aminotransferase
CAT	Catalase
COX-2	Cyclooxygenase-2
CYP	Cytochrome P450
ERK	Extracellular signal regulated kinase
GSH	Glutathione
GPx	Glutathione peroxidase
iNOS	Inducible nitric oxide synthase
JNK	c-Jun N-terminal kinase
Keap 1	Kelch-like ECH-associated protein 1
MAPK	Mitogen activated protein kinase
NAC	*N*-acetylcysteine
NQO1	NADPH:quinone oxidoreductase 1
Nrf2	Nuclear factor erythroid-2-related factor-2
ROS	Reactive oxygen species
SOD	Superoxide dismutase
